# Ethical, legal and social aspects of human cerebral organoids and their governance in Germany, the United Kingdom and the United States

**DOI:** 10.3389/fcell.2023.1194706

**Published:** 2023-11-13

**Authors:** Anja Pichl, Robert Ranisch, Ozan Altan Altinok, Melpomeni Antonakaki, Andrew J. Barnhart, Katherine Bassil, J. Lomax Boyd, Alice Andrea Chinaia, Sarah Diner, Maxence Gaillard, Henry T. Greely, Joshua Jowitt, Karola Kreitmair, David Lawrence, Tim Nicholas Lee, Alex McKeown, Vorathep Sachdev, Silke Schicktanz, Jeremy Sugarman, Katharina Trettenbach, Lara Wiese, Hannes Wolff, Garðar Árnason

**Affiliations:** ^1^ Faculty of Health Sciences Brandenburg, University of Potsdam, Germany; ^2^ Research Unit “Ethics of Genome Editing”, Institute of Ethics and History of Medicine, The University of Tübingen, Tübingen, Germany; ^3^ Center for Ethics and Law in the Life Sciences, University of Hannover, Hannover, Germany; ^4^ Department of Science, Technology and Society, Technical University Munich, Munich, Germany; ^5^ Centre for Biomedical Ethics and Law, KU Leuven, Belgium; ^6^ Department of Psychiatry and Neuropsychology, Maastricht University, Maastricht, Netherlands; ^7^ Berman Institute of Bioethics, Johns Hopkins University, Baltimore, MD, United States; ^8^ IMT School for Advanced Studies, Lucca, Italy; ^9^ Institute for Medical Humanities, Medical Faculty, University of Bonn, Bonn, Germany; ^10^ HYBRIDA Project, University of Oslo, Oslo, Norway and UCLouvain, Louvain-la-Neuve, Belgium; ^11^ Stanford Law School, Stanford University, Stanford, CA, United States; ^12^ Newcastle Law School, Newcastle University, Newcastle, United Kingdom; ^13^ Department of Medical History and Bioethics, School of Medicine and Public Health, University of Wisconsin-Madison, Madison, WI, United States; ^14^ Durham Law School, Durham University, Durham, United Kingdom; ^15^ Edinburgh Infectious Diseases, University of Edinburgh, Edinburgh, United Kingdom; ^16^ Department of Psychiatry, Wellcome Centre for Ethics and Humanities, University of Oxford, United Kingdom; ^17^ Institute of Medical Ethics and History of Medicine, University Medical Center Göttingen, Göttingen, Germany; ^18^ Berman Institute of Bioethics and Department of Medicine, Johns Hopkins University, Baltimore, MD, United States; ^19^ Institute for Social and Health Law, Ruhr University Bochum, Bochum, Germany; ^20^ Chair of Constitutional and Administrative Law, Public International Law, European and International Economic Law, Faculty of Law, University of Passau, Passau, Germany; ^21^ School of Humanities and Social Sciences, University of Akureyri, Akureyri, Iceland

**Keywords:** brain organoid, human cerebral organoids, ELSA, ethics, consciousness, moral status, governance, public engagement

## Abstract

Human cerebral organoids (HCOs) are model systems that enable researchers to investigate the human brain in ways that had previously been impossible. The emergence of HCOs was accompanied by both expert and layperson discussions concerning the possibility of these novel entities developing sentience or consciousness. Such concerns are reflected in deliberations about how to handle and regulate their use. This perspective article resulted from an international and interdisciplinary research retreat “Ethical, Legal and Social Aspects of Human Cerebral Organoids and their Governance in Germany, the United Kingdom and the United States”, which took place in Tübingen, Germany, in August 2022. The retreat focused on whether HCO research requires new ethical and regulatory approaches. It addressed epistemic issues around the detection and theorisation of consciousness, ethical concerns around moral status and research conduct, difficulties for legislation and guidelines managing these entities, and public engagement.

## 1 Introduction

Human cerebral organoids (HCOs) provide unique opportunities for exploring the human brain, its development, and disorders. Researchers now use these increasingly sophisticated models to investigate disease mechanisms, test substances for neurotoxicity or therapeutic effects, and derive patient-specific organoids. Their novelty, complexity, and scope of use raise the question whether research on HCOs requires new ethical and regulatory measures. In this perspective we provide an overview of 1) ethical and epistemic issues such as the theory and detection of consciousness and its relation to moral status as well as 2) issues of research ethics, application and public engagement and 3) governance and patent law. We argue that although new ethical and regulatory measures are not immediately necessary, current ethical and regulatory frameworks face certain limitations and may become inadequate as HCOs are developed further and become larger, more complex and are, in novel ways, connected in assembloids, implanted in animal brains, or combined with computer systems.

## 2 Ethical and epistemic issues concerning consciousness and moral status

Ethical issues regarding HCO research arise against a linguistic and conceptual backdrop that is polymorphous and dynamic. Unsurprisingly, problems of unwarranted expectations and misconceptions arise with the notion of ‘mini-brains’ or ‘brains in a dish’. Even the term ‘organoid’ implies strong similarities regarding features, cell types and functions of the respective organs that are not present in most models. While resemblance to the human brain commonly justifies their use as models in brain research, in ethical discourse, these similarities have raised concerns that HCOs might develop sentience or consciousness. Diner (2023) questions this line of thought, particularly precautionary attempts that rely on resemblance as grounds for caution. The primary reason is that discrepancies among models, as well as a lack of a standard protocol, complicate the identification of the proposed neurological underpinnings of sentience or consciousness in any given HCO. Organoid researchers, at least those interviewed by Lavazza and Chinaia (2023), do not share (some) neuroethicists’ concerns about sentience and moral status of HCOs.

The question of whether a form of consciousness could potentially emerge in HCOs or assembloids poses a theoretical problem as well. Any attempt to assess consciousness requires a theory of consciousness selected for this task. While certain theories give rise to measures that may permit the detection of consciousness in human beings (Casali et al., 2013) none are fit for the biological characteristics of organoids, which might develop conscious states in other ways than humans, thus potentially requiring different tools for assessing consciousness (Diner and Gaillard, 2023). The skepticism prevailing in the neuroscience community regarding the possibility of consciousness-in-a-dish may stem from an assumption, foundational to so-called ‘global theories of consciousness’, wherein consciousness correlates with a global activation of the nervous system. However, there are competing views, so-called ‘local theories’, that suggest that minimal networks can support some form of consciousness. The idea that subsystems responsible for specific tasks could also give rise to conscious states by themselves and in isolation would suggest that consciousness in disconnected, disembodied, disembedded systems may be possible even if its features are difficult to imagine.

Notwithstanding these and other epistemic issues, research on HCOs, as well as assembloids and chimeras involving them, has been accompanied by intensive ethical debates concerning the moral status of these entities and the appropriate ethical protections that consciousness or sentience would bestow upon them (e.g., Farahany et al., 2018; Lavazza and Massimini, 2018; Hyun et al., 2020; Greely, 2021; Niikawa et al., 2022; Sawai et al., 2022). Consciousness alone does not determine the moral status of HCOs according to Kreitmair (2023), who argues that the degree of moral status conferred through consciousness depends on the content of the conscious experience. She rejects the claim that consciousness admits of degrees, which rules out the possibility of a correlation between degree of consciousness and degree of moral status. Since HCOs do not exhibit behavior, the only means for detecting consciousness is through the neural correlates of consciousness (NCCs). However, inference from the NCCs does not provide information fine-grained enough to determine relevant features of the content of conscious states. The upshot is that the ethics of research involving HCOs depends also on value judgments about the degree of tolerance we ought to have for error and uncertainty in determining the moral status of HCOs.

Some hold the view that identifying the conditions under which phenomenal or qualitative consciousness arises in HCOs might even be ethically off limits (McKeown, 2023). If there is success in deliberately deriving conscious organoids in order to understand these conditions, the risk of causing suffering in organoids increases. It follows that researchers ought to only use entities in research where there is ‘sufficient’ confidence that the ‘threshold’ of consciousness has not been reached. This presents a dilemma: On one hand, it is morally problematic to conduct research that could provide insight into morally relevant characteristics, such as consciousness. On the other hand, without such research, the risk persists that experimentation might inadvertently cause organoid suffering, given the lack of knowledge of the conditions for organoid consciousness. Scientific research might have to be conducted, and thus funded, with the primary goal of providing information to help answer ethical questions, such as whether future, much larger, organoids or assembloids could perceive something like pain. This could permit the minimization of harm in further research but faces the above-mentioned epistemological challenges. The moral relevance of consciousness, however, is not primarily a scientific question but a philosophical one, the answer to which must take into account cultural and historical contexts. Current ethical debates on HCOs, in contrast, suffer from an often implicit neuro-centrism. This stance ascribes a decisive role to neuro-somatic development as the basis for an entity’s moral standing, putting aside further moral criteria such as the social meaning given to such entities (Schicktanz, 2020).

## 3 Specific ethical issues concerning research conduct, application scenarios and public engagement

Ethical issues concerning the moral status of organoids, their extent of maturation and provenance, as well as standard ethics considerations for basic and translational research (Bredenoord et al., 2017; De Jongh et al., 2022) relate to organoids in general. They can be distinguished from specific ethical issues that are related to different types of organoid research design which will be briefly touched upon here. There are ethical questions regarding the complexity of experimental systems such as assembloids (Munsie et al., 2017) or the use of HCOs and chimeras for studying stress-related disorders (Bassil and Horstkötter, 2023). Living biobanking encompasses ethical issues associated with the scope of consent, governance, genetic relationships and privacy, and what to do with actionable results (Boers et al., 2016). While there can be broad support among patients for organoid research, acceptable use is predicated on good research intent, oversight and consent as empirical research has shown (Bollinger et al., 2021). However, obtaining consent can be challenging due to a variety of factors, notably including difficulties in accurately communicating the nature of organoids (Sugarman, 2022). Relying on patient-specific organoids with known correlates of efficacy or toxicity in the context of personalized medicine raises ethical issues pertaining to the use of patients’ bodily material and the clinical management of patients under considerable uncertainty (e.g., the possibility of deriving a suitable organoid, time needed for organoid maturation and testing in the face of clinical needs, lack of data on predictability in selecting medications). The prospect of transplanting HCOs necessitates not only considering the risks of interventions into the human brain, but also the difficulty of assessing the functional integration of tissue and its potential side effects on patients’ personality, agency, and sense of identity. If problems of safety and efficacy can be resolved, issues of responsible forms of commercialisation and access to therapies arise (Boers et al., 2018; De Jongh et al., 2022).

The multitude of ethical issues related to HCO research cannot be captured by a single ethical framework but requires a plurality of ethical approaches (Barnhart and Dierickx, 2023a). Exploring HCO research through the lens of the One Health approach (Mackenzie and Jeggo, 2019) may be of interest due to its comprehensive scope and potential to avoid neurocentrism. One Health aims at maximizing human, animal, and environmental health, and places significant importance on social impact. Enshrined in One Health ethics is a moral commitment to animal health which could ground a moral argument for the use of HCOs in order to reduce the number of animals used for research. While uncertainty whether (future) HCOs can suffer prevails, it is generally acknowledged that animals do indeed suffer, and that known suffering should be reduced by shifting away from animal models where feasible. Their replacement with HCOs might also come with scientific advantages of increased reliability of human tissue-derived models that do not pose problems of species differences. HCOs, however, are not only used to replace animal models if their current limitations can be overcome. Indeed, they may create an increased demand for laboratory animals and animal-derived products, given novel human-animal chimera experimentation. Animal ethics frameworks such as the Six Principles framework (DeGrazia and Beauchamp, 2019) might be particularly suitable for exploring ethical issues of HCO research relating to animal welfare and specific ethical issues arising with neural chimeras and xenotransplantation of HCOs (Barnhart and Dierickx, 2023b).

Current understanding of public views toward HCOs is limited to small, fixed-term, and highly local qualitative studies (Haselager et al., 2020; Bollinger et al., 2021; Ravn et al., 2023). Pathways for including public input into the normative analysis of HCO technology are needed, which could help inform subsequent democratic policy recommendations (Boyd and Sugarman, 2022). The development of an open resource for documenting public attitudes, values, and moral evaluations of emerging science and technology such as HCOs might fulfil this purpose. It could provide a structured, scalable, and adaptable framework for documenting and accessing public views on bioethical topics. Obviously, designing automated conversational systems to facilitate massively parallel public engagement raises its own ethical and pragmatic challenges, such as the need to protect the privacy of contributors and how these data are most effectively accessed for subsequent normative analysis and policy evaluations. If these issues can be solved, such a tool Corpus could provide scholars, scientists, and policymakers with greater awareness of public attitudes and values toward emerging neurotechnologies.

The media portrayal of HCO research has been observed to differ from public concerns (Evans, 2022). Media representation of HCOs has been charged with negative or positive exaggerations, especially with regard to the potential emergence of consciousness and therapeutic promises (Presley et al., 2022; Kataoka et al., 2023). This arguably contributes to polarization and undue hope or fear that potentially undermine trust in and support of HCO research. In contrast, little media attention was paid to issues of commercialization, regulation and limitations of model systems (Presley et al., 2022). As an antidote, it has been suggested that neuroethics as a field should play a socio-political role in ‘neurodiscourses’, mediating between broader society and areas such as healthcare, research and neurotechnology (Dubljević et al., 2022). This requires the integration of knowledge about scientific models, consciousness and public imaginaries from areas such as philosophy of science, philosophy of mind, as well as other humanities disciplines (Greely, 2021).

## 4 Governance and patent law

What constitutes appropriate boundaries and regulations for research on HCOs is an open question and the answers may vary within and across different jurisdictions. The International Society for Stem Cell Research (ISSCR) provides guidance toward stem cell researchers worldwide through its regularly updated Guidelines for Stem Cell Research and Clinical Translation. The ethical debates regarding HCO research may have animated the discussions behind the new classificatory schema that introduced a split of category 1 into 1 A and 1B, with the former covering “research determined to be exempt from a specialized … oversight” and applies to all organoid research, while the latter addresses “research that is reportable” (ISSCR, 2021, 10). From a social science perspective, splitting A and B is a testimony to how leaders of the stem cell research community engaged in processes of “building of a consensus around a standardizing framework that establishes a common, global epistemic culture within and through which local research within different labs can be located and valorized.” (Eriksson and Webster, 2015, 73).

In the United States, HCOs are subject to general regulations regarding human subjects research (because of the use of human cells), laboratory animal use (when human-nonhuman chimeras are involved), and stem cells in some cases (e.g., in the case of human embryonic stem cells as source material for HCO derivation in certain jurisdictions or funding sources). Important guidance documents that address these issues are the ISSCR Guidelines, and a report from the National Academies specifically on neural organoids and chimeras (NASEM, 2020). Looking forward, as several of those documents note, new regulatory bodies or forms of oversight may be needed, at national, regional, or international levels. Such groups may well have to govern not only organoids but a wide variety of entities that are not exactly humans or non-humans, embryos or non-embryos, animals or non-animals (Gaillard et al., 2022).

The interdisciplinary working group “Brain Organoids: Opportunities and Limitations” of the German National Academy of Sciences Leopoldina (2022) considers the current legal situation in Germany to be sufficient and suitable for dealing with HCOs in the short and medium term. This conclusion is based on the assumption that for the foreseeable future there is no possibility of the development of organoid consciousness. However, the statement called for a close monitoring of research on HCOs and specialised interdisciplinary ethics commissions for evaluating research proposals that involve the transplantation of HCOs into animals.

If further developed HCOs were to be protected in the same way as animals in German jurisdiction, a change of constitutional law would have to occur according to Wiese (2022). She argues that the introduction of German regulations to protect HCOs for their own sake needs to take into account constitutional law because the derivation and use of HCOs is generally covered by the constitutional commitment to the freedom of science, which, according to the systematics of German law, can only be restricted to the extent necessary to protect other rights or interests of constitutional rank. HCOs, however, neither have (basic) rights themselves, nor is their protection a state objective anchored in the constitution, as is the case with animal protection. Consequently, it is not clear how boundaries could be set on the freedom of science in order to protect HCOs without a change in constitutional law.

Also, the ability of animal law to protect highly evolved mammals faces severe limitations due to their legal status as property in many jurisdictions. In the legal case of Happy,^1^ a 47-year-old elephant, the New York courts accepted that her living conditions at Bronx Zoo without a companion were causally linked to her poor physical and mental health, and that she clearly had a moral claim to redress. Yet, based on the binary legal classification of all things as either persons or things, the courts declined to grant her legal redress (Jowitt, 2023). Under current law, her property status makes her incapable of bearing legal rights, they maintained. In recognising a moral claim it cannot enforce, the law perpetuates an injustice and demonstrates it is deficient according to Jowitt (2023). He argues that legislatures should therefore be proactive in considering the moral status of emerging medical technologies and ensure that this is reflected in their legal status in order to avoid the problem recurring. Precautionary reasoning, which advocates siding with harm avoidance in cases of uncertainty, could therefore not only require scientists to improve their abilities to detect consciousness but also require legislators to ensure that the interests of HCOs are legally protected by appropriate limits on their use as both standalone entities and in chimeras.

In contrast to the reports and statements issued by the National Academies of Sciences in Germany and the United States and the ISSCR, United Kingdom policy has remained quiet on HCOs, notwithstanding their recent history of foresight and relatively rapid responses to other emerging biotechnologies. Laboratories in the United Kingdom are moving ahead with HCO research in an environment of well respected, forward-thinking public bodies and funding organizations such as the Nuffield Council on Bioethics and the Wellcome Trust; and within a wider context of relatively effective governance of emerging biotechnology. This system has proven flexible enough to adapt to controversial developments such as heritable germline gene editing research (Nuffield Council on Bioethics, 2018) and mitochondrial replacement therapies.^2^ It seems surprising then that HCOs have received so little attention in United Kingdom policy.

A highly controversial topic concerning the governance of HCOs, or novel beings more generally (Lawrence and Morley, 2022), is the question of their patentability and possible limitations to it. HCOs in their current state of development are patentable in EU and US jurisdictions. HCOs unproblematically fulfil the general prerequisites of patentability set forth in Art. 3 (1) EU-Directive 98/44/EC (namely, invention, novelty, inventive step, and susceptibility of industrial application). Patentability is excluded in EU patent law if an invention makes use of human embryos or constitutes a stage of the human body in the individual phases of its formation and development. Neither applies to HCOs, unless embryonic stem cells are used for their derivation. Art. 6 (1) EU-Directive 98/44/EC excludes patentability for inventions “where their commercial exploitation would be contrary to *ordre public* or morality.” Apart from hypothetical thought experiments, no scenario comes to mind in which the commercial exploitation of current HCOs violates the *ordre public*. However, the same is not necessarily true for future HCOs. Keeping in mind that a development of consciousness-like abilities cannot be excluded and that an ability for both physical and psychological suffering has been theorized, both of which are aspects of the *ordre public*, certain applications of future HCOs may constitute a violation of the *ordre public* and therefore lead to an exclusion of patentability (Wolff, 2022).

## 5 Discussion

In conclusion, does research on HCOs and chimeras require new ethical approaches and regulatory measures? A clear and simple answer is not in sight, as the evaluation of these novel entities is confronted with the aforementioned conceptual, theoretical and practical issues. While new technologies such as organoids do not necessarily demand new regulation and new ethical approaches, there are problems and gaps in existing ethical and legal frameworks that warrant attention such as the lack of actual legal protection for highly developed mammals that puts the debate about the moral and legal status of HCOs in perspective. Furthermore, current ethical and legal frameworks may no longer be sufficient to deal with increasingly complex HCOs or issues arising from their connection in assembloids, with computer systems, and their transplantation for research or medical purposes. Therefore, a consensus prevailed among the retreat participants that ethical issues, including those that still appear remote, need to be proactively addressed. At the same time, ethicists need to take care to limit their engagement with speculative scenarios which is most problematic when playing a role in science policy development or governance. Anticipation of potential future developments and ethical concerns should also not divert attention from allegedly mundane, but perhaps rather immediately impactful issues such as commercialisation, the handling of research animals, and access to potential therapies.

## Data Availability

The original contributions presented in the study are included in the article/Supplementary Material, further inquiries can be directed to the corresponding author.

## Footnotes

1 Cf. The Nonhuman Rights Project NhRP Inc (On behalf of Happy) v. Breheny and Wildlife Conservation Society. Index No: 18–45164; available at: https://www.nonhumanrights.org/content/uploads/Happy-Petition-10.1.18.pdf.

2 Cf.: The Human Fertilisation and Embryology (Mitochondrial Donation) Regulations 2015, N. 572 (2015): https://www.legislation.gov.uk/ukdsi/2015/9780111125816/contents.
